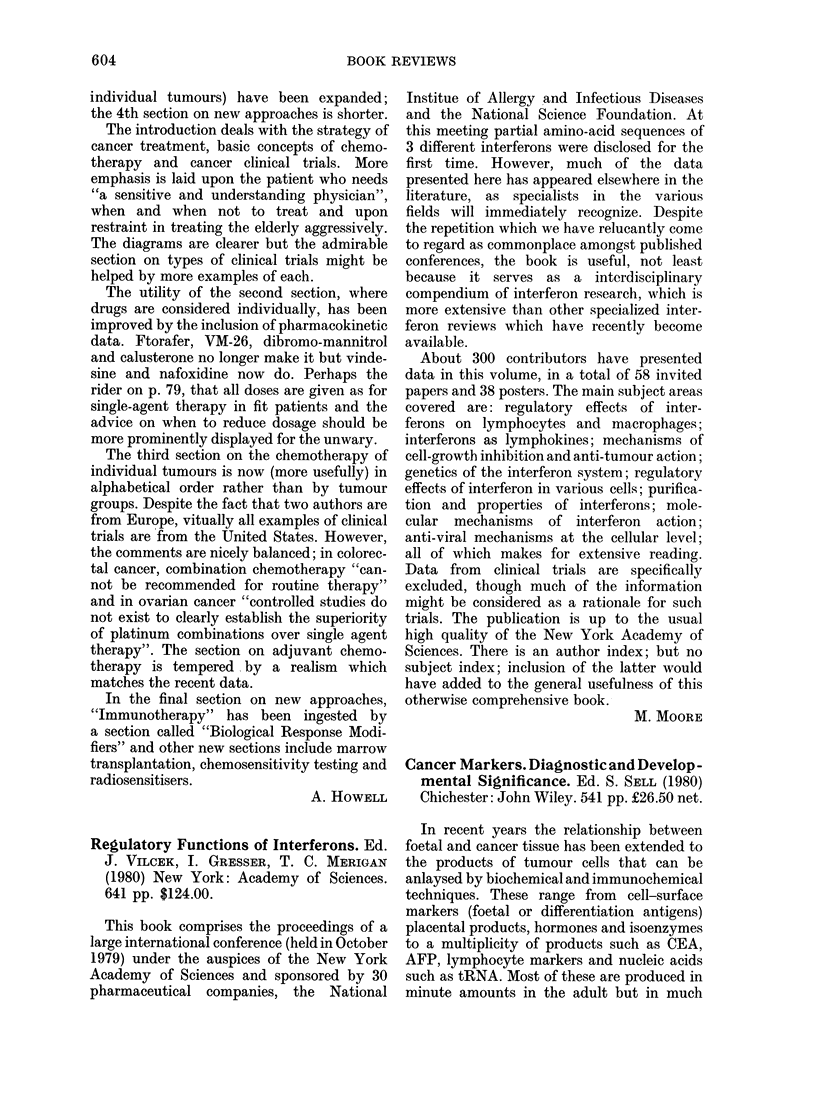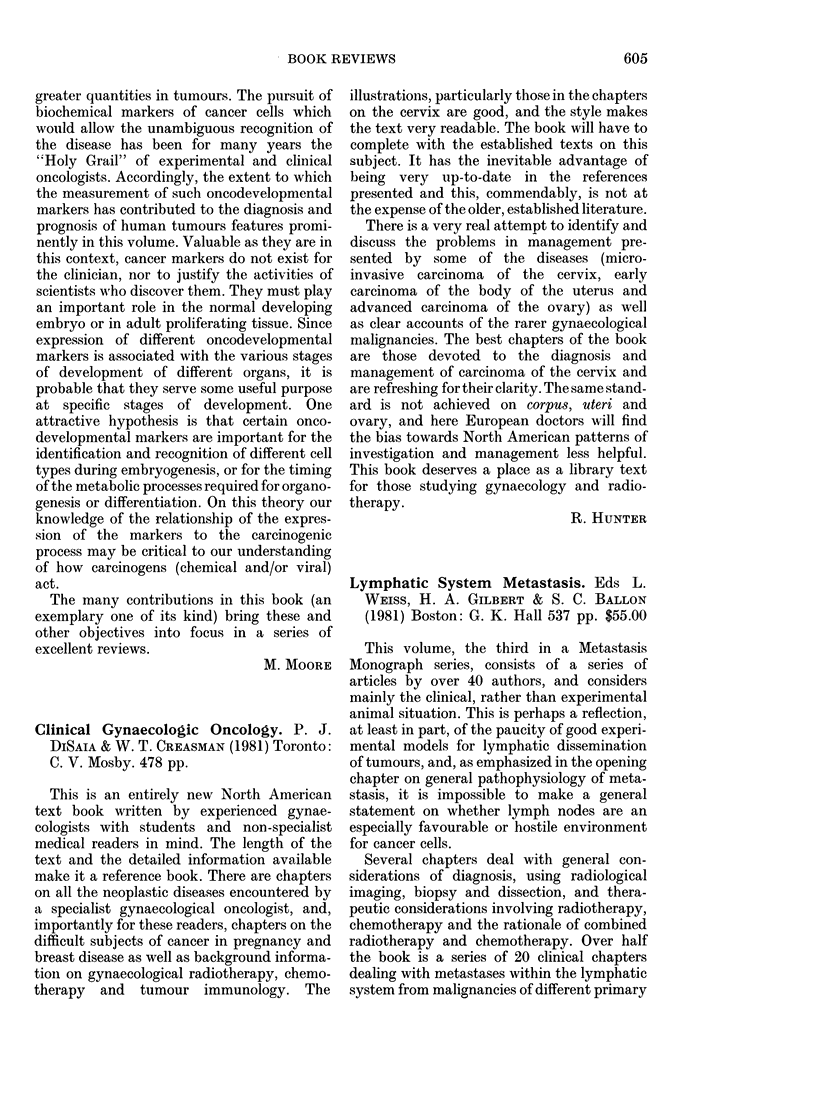# Cancer Markers. Diagnostic and Developmental Significance

**Published:** 1981-10

**Authors:** M. Moore


M. MOORE

**Cancer Markers. Diagnostic and Developmental Significance.** Ed. S. SELL (1980) Chichester: John Wiley. 541 pp. £26.50 net.

In recent years the relationship between foetal and cancer tissue has been extended to the products of tumour cells that can be anlaysed by biochemical and immunochemical techniques. These range from cell–surface markers (foetal or differentiation antigens) placental products, hormones and isoenzymes to a multiplicity of products such as CEA, AFP, lymphocyte markers and nucleic acids such as tRNA. Most of these are produced in minute amounts in the adult but in much greater quantities in tumours. The pursuit of biochemical markers of cancer cells which would allow the unambiguous recognition of the disease has been for many years the "Holy Grail" of experimental and clinical oncologists. Accordingly, the extent to which the measurement of such oncodevelopmental markers has contributed to the diagnosis and prognosis of human tumours features prominently in this volume. Valuable as they are in this context, cancer markers do not exist for the clinician, nor to justify the activities of scientists who discover them. They must play an important role in the normal developing embryo or in adult proliferating tissue. Since expression of different oncodevelopmental markers is associated with the various stages of development of different organs, it is probable that they serve some useful purpose at specific stages of development. One attractive hypothesis is that certain oncodevelopmental markers are important for the identification and recognition of different cell types during embryogenesis, or for the timing of the metabolic processes required for organogenesis or differentiation. On this theory our knowledge of the relationship of the expression of the markers to the carcinogenic process may be critical to our understanding of how carcinogens (chemical and/or viral) act.

The many contributions in this book (an exemplary one of its kind) bring these and other objectives into focus in a series of excellent reviews.

M. Moore